# The global reach of artificial intelligence in atopic dermatitis: The quality and reliability of ChatGPT responses in 8 languages

**DOI:** 10.1016/j.jdin.2024.08.013

**Published:** 2024-09-15

**Authors:** Pranvera Sulejmani, Olivia Negris, Valeria Aoki, Helène Aubert, Chia-Yu Chu, Mette Deleuran, May El Hachem, Lawrence Eichenfield, Ana Mosca, Raquel Leão Orfali, Marketa Saint Aroman, Jean-Francois Stalder, Alain Taieb, Antonio Torrelo, David Troya, Magdalena Trzeciak, Christian Vesttergaard, Andreas Wollenberg, Peter Lio

**Affiliations:** a Department of Internal Medicine, Saint Francis Hospital, Evanston, Illinois; b Department of Internal Medicine, NorthShore University HealthSystem, Evanston, Illinois; c Faculdade de Medicina FMUSP, Departament of Dermatology, Universidade de São Paulo, São Paulo, São Paulo, Brazil; d Dermatology Department, Nantes Université, CHU Nantes, Nantes, France; e Department of Dermatology, National Taiwan University Hospital and National Taiwan University College of Medicine, Taipei, Taiwan; f Department of Dermatology and Venereology, Aarhus University Hospital, Aarhus, Denmark; g Dermatology Unit and Genodermatosis Unit, Translational Paediatrics and Clinical Genetics Research Division, Bambino Gesù Children's Hospital, IRCCS, Rome, Italy; h Departments of Dermatology and Pediatrics, University of California, San Diego and Rady Children's Hospital-San Diego, San Diego, California; i Hospital Municipal Jesus/Hospital Universitário, Pedro Ernesto/Universidade do Estado do Rio de Janeiro, Rio De Janeiro, Brazil; j Medical Direction Pharma, Dermocosmetics Care & Personal Care, Pierre Fabre, Toulouse, France; k Dermat ology Department, Université de Bordeaux, Bordeaux, France; l Department of Dermatology, Hospital Infantil Niño Jesús, Madrid, Spain; m Nantes Université, CHU Nantes, École Centrale Nantes, Nantes, France; n Department of Dermatology, Venereology and Allergology, Medical University of Gdansk, Gdansk, Poland; o Department of Dermatology and Allergy, University Hospital Augsburg, Augsburg, Germany; p Department of Dermatology and Allergy, Ludwig-Maximilian University of Munich, Munich, Germany; q Department of Dermatology, Feinberg School of Medicine, Northwestern University, Chicago, Illinois

*To the Editor:* Atopic dermatitis (AD) is the most common chronic inflammatory skin condition with a staggering global burden of disease. The epidemiology of AD is highly variable worldwide and most of the published literature has so far focused mainly on Western populations. The demand for health care is increasing globally with notable disparities in access to resources, especially in Asia, Africa, and Latin America.^1^ The rapid expansion of artificial intelligence (AI) has the potential to revolutionize health care.^2^^,^^3^ The previous paper published by our group presented an initial assessment of AI-generated responses to common patient queries, focusing on ChatGPT-4's performance.^4^ The findings indicate that ChatGPT-4 offers comprehensive and reliable answers for English language queries. Although ChatGPT appears to perform well in English, it is critical to conduct comparative research in other languages.

This study seeks to offer a follow-up evaluation of the quality and reliability of the responses generated by ChatGPT-4 patient questions related to AD in 8 different languages.

One hundred-one commonly asked questions from AD patients were submitted to ChatGPT-4 in 8 languages. Dermatologists proficient in these languages evaluated the generated responses for quality and reliability and assigned scores to the responses based on a 5-point Likert scale. The graded responses were averaged and ranged from 3.98 to 4.62, indicating variability across languages. Table I presents the average pertinence score ratings for each of the 8 languages evaluated.

**Table I:** Average pertinence score ratings for each of the 8 languages evaluated

Language	Average pertinence score (1-5)
Brazilian Portuguese	3.98
Chinese	4.23
Spanish	4.24
Polish	4.31
German	4.33
French	4.33
Danish	4.44
Italian	4.62

The results indicate that while ChatGPT provides useful information, there are significant variations in response quality across languages, and certain medical topics require improved accuracy and comprehensiveness. Common themes from evaluators included that ChatGPT recognized the need for medical consultation but lacked detailed responses to specific questions on treatments and conditions.

Additionally, the large language model defaulting to “may be helpful” statements in instances where there is a known lack of evidence-based medicine, such as with alternative or traditional Chinese medicine, is problematic. Modern guidelines would state that data-driven medicine is lacking and thus recommend against using said interventions. Specific to the Chinese language, discrepancies in responses with low scores were not caused by limited language proficiency, but rather by the abundance of online resources about traditional Chinese medicine and probiotics. ChatGPT gathers information from the internet and incorporates it into responses, regardless of accuracy. Thus, it is important to recognize that this linguistic localization may introduce significant differences due to the prevalence of information (and misinformation) within a given language sphere.

Noticeable discrepancies exist in ChatGPT’s response evaluation across languages, with some responses being highly relevant and others deemed irrelevant. The five lowest-scoring questions in languages other than English were rated highly (9.09-10/10) by international dermatologists when reviewed in English. This implies that AI-generated responses might not be as accurate in other languages. In conclusion, the large language model's effectiveness varies across languages, and this inconsistency does not follow a clear pattern. Extra caution is advised when using AI in languages other than English, as ChatGPT-4, while suitable for obtaining factual information, isn’t fully equipped to address common medical questions, especially outside of English.

## Conflicts of interest

Dr Aoki has been an investigator and/or consultant to Abbvie, Eli Lilly, and Pfizer. Dr Orfali has been an investigator and/or consultant for Bayer, Eli Lilly, Abbvie, Sanofi, and Amgen. Dr Saint Aroman is an employee of Laboratoires Pierre Fabre, France. Dr Trzeciak has been a speaker and/or consultant and/or investigator and/or participant of Advisory Board for Abbvie, Bausch Health, Bioderma, Eli Lilly, La Roche-Posay, Leo Pharma, Mead Johnson, Novartis, Pfizer, Pierre Fabre, Pfizer, Mead Johnson, and Sanofi Genzyme. Dr Lio reported receiving grants from AOBiome, Regeneron/Sanofi Genzyme, and AbbVie; personal fees from Regeneron/Sanofi Genzyme, Leo, Eli Lilly, Pfizer, Galderma, L’Oreal, Almirall, ASLAN Pharma Advisory board, Dermavant, Pierre Fabre, Menlo Therapeutics, IntraDerm, Exeltis, AOBiome, Arbonne, and Amyris; stock options from Micreos; and other royalties from patented product from Theraplex. Drs Sulejmani, Negris, Aubert, Chu, Deleuran, El Hachem, Eichenfield, Mosca, Stalder, Taieb, Torrelo, Troya, Vesttergaard, and Wollenberg have no conflicts of interest to declare.
